# Quantum Purity as an Information Measure and Nernst Law

**DOI:** 10.3390/e25081113

**Published:** 2023-07-26

**Authors:** F. Pennini, A. Plastino

**Affiliations:** 1 Departamento de Física, Universidad Católica del Norte, Av. Angamos 0610, Antofagasta 1270709, Chile; fpennini@ucn.cl; 2 Departamento de Física, Facultad de Ingeniería, Universidad Nacional de Mar del Plata (UNMDP), CONICET, Mar del Plata 10850, Argentina; 3 Instituto de Física La Plata—CCT-CONICET, Universidad Nacional de La Plata, C.C. 727, La Plata 1900, Argentina

**Keywords:** Nernst law, statistical mechanics, purity

## Abstract

We propose to re-express Nernst law in terms of a suitable information measure (IM) parameter. This is achieved by dwelling on the idea of adapting the notion of purity in the case of a thermal Gibbs environment, yielding what we might call the “purity” indicator (which we denote by the symbol *D* in the text). We find it interesting to define an extension of this D−IM indicator in a classical context. This generalization turns out to have useful conceptual consequences when used in conjunction with the classical Shannon entropy *S*. Implications for the Nernst law are discussed.

## 1. Introduction

In quantum mechanics, purity measures how similar a given quantum state is to a pure one. In other words, it provides insights into the degree to which a quantum state is pure or mixed, and hence the amount of information that can be extracted from it. Mathematically, the purity *D* of a quantum state is given by the trace of the square of its density operator ρ, i.e., $Tr(\rho^{2})$. For a pure state, the purity is 1, indicating that the state is entirely non-mixed. On the other hand, for a maximally mixed state (also known as a completely mixed state), the purity is 1/M, where *M* is the dimensionality of the Hilbert space of the system. In a mixed state, the purity lies between these two extremes.

Both the purity and degree of mixture 1−D provide measures of the information content present in a quantum state. Pure states, with a purity of 1, typically exhibit a coherent superposition and can be entangled. Mixed states, on the other hand, have reduced purity and contain a mixture of quantum states with varying probabilities. The degree of mixture quantifies the extent of the mixture and reflects the amount of information and entanglement present in the state. These information measures are widely used in quantum information theory, quantum state estimation, and the characterization of quantum systems, as they provide valuable insights into the purity and degree of mixture of quantum states [1,2,3,4,5,6].

### Our Goals and Organization

The third law of thermodynamics (Nernst law) implies that it is impossible to reach absolute zero temperature (AZT) by any finite number of steps, and that at AZT the entropy of a perfectly ordered crystalline substance vanishes. In other words, a perfectly ordered crystal at absolute zero temperature would have no disorder or randomness, and would be in a state of minimum energy [7].

This law has important implications for the behavior of matter at low temperatures and is essential for understanding the behavior of condensed matter physics, including the properties of solids, liquids, and superfluids. The third law is also used in the design and optimization of refrigeration and cooling systems, as well as in the study of chemical reactions and the behavior of materials under extreme conditions [7].

Here, instead, we discuss an alternative viewpoint. We will try to embark on a discussion of information theory in which the protagonist is the notion of purity *D*, regarded as an information measure (IM).

We will focus our attention on the harmonic oscillator (HO) and on the ideal gas to provide an understandable and clear picture of the situation. The simplicity of the systems enables one to more easily visualize what the alternative use of the parameter *D* allows to be uncovered in an actual physical situation.

Thus, we will try to link the third law of thermodynamics to the notion of purity within the framework of (1) the harmonic oscillator and (2) the ideal gas. More details on this are as follows:

- In Section 2, we consider the purity notion first in a quantum thermal scenario and then in a classical environment.
- In Section 3, we obtain a useful expression for the purity quantifier *D* that relates it to the Helmholtz free energy. This relation serves to considerably simplify our *D* manipulations later.
- In Section 4, we consider the quantum harmonic oscillator (HO) and devise a spatial method for expressing its Shannon entropy *S* solely in *D* terms, which illuminates some aspects of Nernst law.
- In Section 5, the nucleus of this effort, we consider the classical HO and comment on some interesting traits.
- Section 6 is devoted to the ideal gas. Some new insight is gained.
- Section 7 is devoted to conclusions.

## 2. A Generalization of the Purity Notion to a Finite Temperature Scenario

The density operator that describes the statistical ensemble of quantum states in a system at finite temperature incorporates the probabilities associated with different energy eigenstates of the system. In the case of a discrete set of energy eigenstates, the density operator can be expressed as

(1) $$\rho =\sum_{i}P_{i}\;|i\rangle \langle i|,$$

where $P_{i}$ represents the probability associated with the *i*-th energy eigenstate. It is determined by the thermal equilibrium, a canonical distribution of Gibbs’ that uses the so-called exponential Boltzmann factors [7]

(2) $$P_{i}=\left(1/Z\right)\exp(-\beta E_{i}),$$

so that our principal system input is the set of level energies $E_{i}$. We denote β=1/kT (*k* is the Boltzmann constant). This entails a set of canonical probabilities $P_{i}=\exp\left(-\beta E_{i}\right)/Z$, *Z* being the partition function. Several quantifiers like the entropy *S* or the free energy *F* are built up with the $P_{i}$ [7]. By summing over all possible energy eigenstates and incorporating the corresponding probabilities, the appropriate density operator captures the ensemble average behavior of the system at finite temperature. The diagonal elements of the density operator provide the populations of the energy eigenstates, while the off-diagonal elements capture the coherence and quantum correlations between different eigenstates. The density operator at finite temperature provides a powerful tool for analyzing and characterizing the behavior of quantum systems under thermal equilibrium conditions [7], accounting for the probabilities associated with different energy eigenstates and their corresponding energies. The purity, *D*, is obviously

(3) $$D=\mathrm{Tr}\rho^{2}=\sum_{i}P_{i}^{2}.$$

Notice that small *D* might be said to entail a kind of disorder, as we have only very low probability elements, $P_{i}$, that can not differ too much from one another—that is, we are very close to a uniform distribution representing maximum randomness. On the other hand, large *D* might be said to imply order as one must encounter some (relatively) very large $P_{i}$s, indicating preferred microstates and thus some kind of structure. Thus, *D* might be regarded as an order–disorder indicator. The classical counterpart of *D*, to be discussed below, is known to be an indicator of such kind.

### Generalization of the Purity Notion to a Classical Scenario

A pertinent point can be made in terms of a statistical concept developed around 25 years ago, called the disequilibrium, an order–disorder quantifier [8]. Low disequilibrium entails disorder and large disequilibrium entails order.

The central idea is that, statistically, maximum disorder is represented by a uniform distribution (UF) [8]. As a consequence, the more different our current probability distribution (CPD) is from the UF, the more order this CPD represents [8]. The associated Euclidean distance in the probability space between the CPD and the UF is the disequilibrium [8] $Q=\sum_{i}P_{i}^{2}$, which defines a quantity *Q* that is identical to the one called *D* above for a different purpose. The product of *Q* with the Shannon entropy

(4) S=−Trρlnρ

is of course the thermodynamic entropy [7].

(5) C=QS=DS,

is called the statistical complexity. This expression of *C* is in many instances the standard complexity form employed in several current publications. For a small sample, see, for instance, Refs. [9,10,11,12,13,14,15,16]. Why is repeating the symbol *D* for naming two different notions adequate? Because, formally, both equal the sum of the squares of probability elements $P_{i}$. At a finite temperature and within Gibbs’ canonical ensemble, two pertinent types of probability coincide.

## 3. Relation between D and the Helhmoltz Free Energy F

Given the partition function *Z*, we have the sequence [7]

(6) $$Z\left(T\right)=\sum_{i}\exp\left(-\beta E_{i}\right),$$

(7) F(T)=−kTln(Z(T)),

(8) F(T/2)=−k(T/2)ln(Z(T/2)),

(9) $$2\beta \left[F\left(T\right)-F\left(T/2\right)\right]=2\beta kT\left[(1/2)\ln\left(Z(T/2)\right)-\ln\left(Z(T)\right)\right],$$

(10) $$2\beta \left[F\left(T\right)-F\left(T/2\right)\right]=\ln\left[\frac{Z(T/2)}{Z{\left(T\right)}^{2}}\right],$$

(11) $$\exp\left(2\beta [F(T)-F(T/2)]\right)=\frac{Z(T/2)}{Z{\left(T\right)}^{2}},$$

(12) $$\frac{Z(T/2)}{Z{\left(T\right)}^{2}}={\left[\sum_{i}\exp(-\beta E_{i})/Z\right]}^{2}=\sum_{i}P_{i}^{2}=D,$$

so that we finally arrive at our desired relation that connects *D* with *F*:

(13) D=exp[2β(F(T)−F(T/2))].

We will use this important expression in the following sections.

## 4. Purity and the Quantum Harmonic Oscillator

Consider three-dimensional harmonic oscillators of frequency ω in equilibrium at temperature *T*, whose energies are quantified according to $\epsilon_{n}=\hbar \omega \left(n+3/2\right)$, with n=0,1,2,… The canonical partition function for a single oscillator is [7]

(14) $$Z_{1}=\frac{e^{-3\beta \hbar \omega /2}}{{\left(1-e^{-\beta \hbar \omega }\right)}^{3}},$$

while the Shannon entropy reads [7]

(15) $$S=3k\left[\frac{\beta \hbar \omega \;e^{-\beta \hbar \omega }}{1-e^{-\beta \hbar \omega }}-\ln\left(1-e^{-\beta \hbar \omega }\right)\right],$$

which is definite positive for all temperatures *T*. Also, the Helmholtz free energy is

(16) $$F=\frac{3}{2}\hbar \omega +3kT\ln\left(1-e^{-\beta \hbar \omega }\right).$$

Now, we follow the indications given in Ref. [9] in order to obtain the purity *D* that, as we just saw above, is a simple function of the canonical Helmholtz free energy *F*. In Equation (13), for the purity, we had

(17) D=exp[2β(F(T)−F(T/2))].

Therefore, considering that *F* is given by Equation (16), then one finds that the purity is given by

(18) $$D={\left(\frac{1-e^{-\beta \hbar \omega }}{1+e^{-\beta \hbar \omega }}\right)}^{3}=\tanh^{3}\left(\frac{\beta \hbar \omega }{2}\right).$$

Note that when T=0, then D=1, and for *T* tending towards infinity, we have D=0. Therefore, the purity is of course bounded, i.e.,

(19) 0≤D≤1.

The above relation (18) shows that for small β the hyperbolic tangent above approaches βℏω/2. From Equation (18), we can isolate the variable βℏω in the fashion

(20) $$\beta \hbar \omega =-\ln\left(\frac{1-D^{1/3}}{1+D^{1/3}}\right)=2\;\mathrm{arctanh}\left(D^{1/3}\right).$$

Now, introducing Equation (20) into Equation (15), we find the entropy *S* can be cast as a function of just *D* [17]. Thus, we reach the wished-for possibility of expressing the entropy solely in terms of the purity

(21) $$S=-3k\left[\left(\frac{1-D^{1/3}}{2D^{1/3}}\right)\ln\left(\frac{1-D^{1/3}}{1+D^{1/3}}\right)+\ln\left(\frac{2D^{1/3}}{1+D^{1/3}}\right)\right].$$

We clearly see that maximum *D* yields zero entropy, and the third law of thermodynamics can be applied in terms of purity. Let us insist on this point: *When we express S in terms of D, we realize that, at maximum purity, the entropy vanishes. This may seem rather obvious, but it opens the door for us to ask: what happens classically when we express the classical S in terms of the classical D?* Could it be that some similar *S*-*D* connection might emerge? We will see below that the answer is a positive one.

## 5. Classical Harmonic Oscillator and Classically Generalized D

We saw above that the classically generalized *D* is called disequilibrium, a notion developed some 25 years ago, independently of the purity idea [8]. The connection between purity and disequilibrium is being made in this article. Let us then consider the three-dimensional harmonic oscillators of frequency ω in a Gibbs canonical ensemble at the temperature *T*, whose respective Hamiltonian is $H=\sum_{i=1}^{3N}p_{i}^{2}/\left(2m\right)+m\omega^{2}x_{i}^{2}/2$ [7]. *We wish to ascertain what happens when we attempt to express S in terms of D*.

The partition function of each oscillator is

(22) $$Z={\left(\frac{kT}{\hbar \omega }\right)}^{3},$$

and contains all the statistical HO information necessary for our ends. The entropy is [7]

(23) $$S=3k\left[1-\ln\left(\frac{\hbar \omega }{kT}\right)\right],$$

which is positive definite whenever

(24) T≥ℏω/(ek).

This yields the condition to avoid negative entropies.

Remember that we saw above that *D* is a simple function of *F*, of a form given by Equation (17) [9]. Then, considering that F=−kTlnZ, one finds

(25) $$D={\left(\frac{\hbar \omega }{2kT}\right)}^{3}.$$

and also

(26) $$Z=\frac{1}{2D},$$

which assures us that the whole classical HO statistical mechanics is governed by *D*.

Remark that, as in the quantum instance, both the partition function and *S* are simple functions of *D* and, importantly enough (see Equations (24) and (25)),

(27) $$0\leq D\leq D_{max}.$$

In addition, the classical statistical complexity is of the form

(28) $$C=D\left(S/k\right)=3{\left(\frac{\hbar \omega }{2kT}\right)}^{3}\left[1-\ln\left(\frac{\hbar \omega }{kT}\right)\right].$$

### Present Results for the HO

Let us first (a) insist that the generalized *D* is a measure of order, and (b) repeat Equation (25)

(29) $${\left(\frac{\hbar \omega }{2kT}\right)}^{3}=D,$$

to emphasize that **the generalized purity, here called disequilibrium, is the ratio between the HO’s vibrational and kinetic energies.** Replacing this into Equation (28), we are led, for the single HO, to the desideratum of the expression of the Shannon entropy entirely in terms of *D*, i.e.,

(30) $$S=3k[1-\ln\left(2D^{1/3}\right)].$$

It is clear that the entropy will vanish for a special *D* value. We need to ascertain the meaning of such special value. Now, for the statistical complexity, we find

(31) $$C=3D\left[1-\ln(2D^{1/3})\right].$$

It is important now to note that, given that the *C* measure must be a positive quantity, as stipulated in Ref. [18], then the maximum classical value for *D* can not be infinite. It must at most adopt that value which makes $\ln(2D^{1/3})=1$, entailing, as we saw above and repeat here because of its relevance,

(32) $$0\leq D\leq D_{max},$$

with

(33) $$D_{max}={\left(e/2\right)}^{3}∼2.50.$$

This provides us with a maximal classical degree of order. Also, it results in several interesting and hopefully new classical features. The *C*-positivity argument also applies to the entropy, of course, with identical results.

*Additionally, for such a $D_{max}$—disequilibrium value—the entropy vanishes according to Equation (30). Thus, maximum order implies zero entropy as in the quantum instance above, which can be rephrased as an order-based third law.* The special quantum connection *S*-*D* of the preceding section has indeed a classical counterpart. We can thus loosely speak of a kind of classical Nernst law of vanishing entropy at the situation of maximum order (as measured by *D*).

Let us here insist again on a previous assertion by reiterating that from Equation (25) we saw that

(34) $$\frac{\hbar \omega }{2kD^{1/3}}=T,$$

The temperature grows as the degree of disorder increases, as is intuitively obvious. But, by now using Equation (33), we see that this last assertion implies that as the order, which grows with *D*, has a maximum, the classical temperature has a frequency-dependent (non zero!) minimum (see Equation (36) below). For

(35) $$D>{\left(e/2\right)}^{3}\;\;\;,\mathrm{one}\mathrm{finds}\;\;\;S<0.$$

**Negative entropy entails using an incorrect value for *D*** or, in the same way, a too-low energy, according to the prescription $u<u_{minimum}$ with

(36) $$u_{minimum}=\frac{\hbar \omega }{e}.$$

Moreover, we realize that

(37) $$\beta =\frac{2D^{1/3}}{\hbar \omega },$$

so the Gibbs canonical probability *P* for an HO Hamiltonian H and a partition function *Z* reads

(38) $$P=\frac{\exp(-\frac{2D^{1/3}}{\hbar \omega }H)}{Z},$$

which tells us that the HO physics is fully determined by the ratio $D^{1/3}/\omega$. We duly note that *D* has a maximum possible value. Given Equations (33) and (34), we discover that, for a fixed frequency (neither too high nor too low) the permissible minimum temperature is of the order of $10^{-10}$ Kelvin. As an additional comment, we revisit the mean energy of the harmonic oscillator [7]

(39) u=3kT.

Thus, in view of Equation (29), for the single HO, we have, for *N* HOs, a mean energy *U*

(40) $$u=\frac{3\hbar \omega /2}{D^{1/3}},$$

The above classical (and of statistical origin) minimum energy has a value slightly smaller that the well-known quantum one.

We see that the HO energy can be thought of as being purely originating from disorder. If the order degree indicator D is large enough, the system will be encountered at a very low energy, but not arbitrarily low. This is indeed a new result. The classical HO seems to have a minimum energy, a minimum temperature, and a maximal order degree at which S=0, if we respect the condition that the statistical complexity cannot be negative.

## 6. The Ideal Gas

The system consists of *N* mono-atomic identical particles contained in a volume *V*. They are in thermal equilibrium at temperature *T*. The pertinent Hamiltonian reads $H=\sum_{i=1}^{N}\;p_{i}^{2}/2m$, where *m* is the mass of the particles and $p_{i}$ the concomitant momenta, with i=1,…,N [7,19]. The associated canonical partition function takes the form [7,19]

(41) $$Z_{N}=\frac{1}{N!}\;{\left(\frac{V}{\lambda^{3}}\right)}^{N}.$$

$\lambda =h/\sqrt{2\pi mkT}$ stands for the particles’ mean thermal wavelength [7]. The Helmholtz free energy *F* can be cast as [7]

(42) $$F=NkT\;\left[\ln\left(n\;\lambda^{3}\right)-1\right],$$

with n=N/V and v=V/N being the molar density and volume per particle [7].

The classical entropy is provided by the well known Sackur–Tetrode equation that reads [7]

(43) $$S=Nk\ln\left[e^{5/2}\;{\left(n\;\lambda^{3}\right)}^{-1}\right],$$

which is positive definite if we fulfill the requirement $n\;\lambda^{3}\ll e^{5/2}$.

### The S-D Connection

According to Ref. [19], one has for *D* and the complexity *C* per particle

(44) $$D=n\;\lambda^{3}\;e^{-1}\;2^{-3/2},$$

and then

(45) $$C=n\;\lambda^{3}\;e^{-1}\;2^{-3/2}\;\ln\left[e^{5/2}\;{\left(n\;\lambda^{3}\right)}^{-1}\right].$$

Remark that λ is the thermal de Broglie wavelength, that is, roughly, the mean de Broglie wavelength of the gas molecules at the temperature *T*. The mean inter-particle spacing $d_{T}$ is, approximately, $v^{1/3}$. Whenever λ is much smaller than this $d_{T}$, the gas can be regarded as classical. Contrarily, when λ is of the order of or larger than $d_{T}$, quantum effects should dominate and the gas ought to be treated as quantal.

Notice that, on the basis of the above relations, both *S* and *C* become negative for $n\lambda^{3}>e^{5/2}$, which involves $D>{\left(e/2\right)}^{3/2}\;∼\;1.59$. If we wish to avoid this negativity, then there is a maximum degree of order D=1.59, an important result. Clearly, for D=1.59 the complexity C=0. A simple manipulation yields the mean energy u=U/N for D=1.59 as

(46) $$u_{minimum}=\frac{6\pi \hbar^{2}}{mv^{2/3}e^{5/3}},$$

a very small but non-zero quantity. Thus, in these circumstances, we can speak of a maximum degree of order for which the entropy vanishes—an order-related Nernst law.

## 7. Conclusions

What this paper offers is a choice regarding the nature of the classical entropy *S*. We can either accept the possibility of having S<0, as most people seem to do, or reject it. If we reject it, then the degree of order exhibits, at its maximum, at which S=0, an order-based Nernst law.

Based on statistical mechanics considerations on the notion of purity and its quantum counterpart (both symbolized by the same letter *D*), we have developed some interesting, and hopefully new, classical traits of the harmonic oscillator and of the ideal gas. They derive from the fact that a complexity measure cannot be a negative quantity [18]. This perhaps also be said of *S*. In our discussion, the key role is played by the notion of purity disequilibrium *D*.

On such a basis, in both the classical HO and ideal gas statistics, the traits described below became apparent.

- Quantum purity is an indicator of how different a quantum state is from the totally mixed one. Its classical counterpart is called disequilibrium and is an indicator of how different the classical probability distribution is from the uniform one. We are talking of the same idea as expressed in two quite distinct scenarios. Notably, the same mathematical expression, which we call *D*, can be used in the two contexts.
- *D* has a maximum possible value. In the quantum case, this maximum value is unity. Classically, we saw in two examples that there is a maximum value $D_{max}={\left(e/2\right)}^{3}$ for the HO and $D_{max}={\left(e/2\right)}^{3/2}$ for the ideal gas.
- We conjecture that, classically, the maximum value of *D*, which can be thought to represent maximum order, is equal to ${\left(e/2\right)}^{f/2}$, with *f* being the number of degrees of freedom of the system. For a three-dimensional HO f=6, and for the ideal gas f=3.
- For such maximum order values, the classical HO entropy and the ideal gas one vanish at a temperature of $T_{0}$ (with $T_{0}\neq 0$), a kind of order0based third law that applies not at zero temperature but at a finite one.
- There exist minimum possible classical mean energy (HO and ideal gas) values. This seems to be a new and surprising result.
- Either the classical HO or the ideal gas entropies are negative for a $D>D_{maximum}$ situation. Since we do not want a negative Shannon entropy, then *D* cannot exceed D(maximum).
- If we declare that there are only positive entropy values, this fact by itself prevents the classical temperature from reaching the zero value, as we have already established by appealing to conventional treatments.
- We remark that our approach does not seem to require the concept of negentropy advocated by the Prigogine school [20].

## Data Availability

Everything that might be needed is here.
